# Harnessing Recent Advances in Synthetic DNA and Electroporation Technologies for Rapid Vaccine Development Against COVID-19 and Other Emerging Infectious Diseases

**DOI:** 10.3389/fmedt.2020.571030

**Published:** 2020-10-21

**Authors:** Ziyang Xu, Ami Patel, Nicholas J. Tursi, Xizhou Zhu, Kar Muthumani, Daniel W. Kulp, David B. Weiner

**Affiliations:** ^1^The Vaccine and Immunotherapy Center, Wistar Institute, Philadelphia, PA, United States; ^2^Department of Pharmacology, Perelman School of Medicine, University of Pennsylvania, Philadelphia, PA, United States

**Keywords:** DNA vaccines, intradermal electroporation, coronaviruses, COVID-19, SARS-CoV-2, emerging infectious diseases (EIDs), DNA-launched nanoparticle vaccines, intranasal vaccines

## Abstract

DNA vaccines are considered as a third-generation vaccination approach in which antigenic materials are encoded as DNA plasmids for direct *in vivo* production to elicit adaptive immunity. As compared to other platforms, DNA vaccination is considered to have a strong safety profile, as DNA plasmids neither replicate nor elicit vector-directed immune responses in hosts. While earlier work found the immune responses induced by DNA vaccines to be sub-optimal in larger mammals and humans, recent developments in key synthetic DNA and electroporation delivery technologies have now allowed DNA vaccines to elicit significantly more potent and consistent responses in several clinical studies. This paper will review findings from the recent clinical and preclinical studies on DNA vaccines targeting emerging infectious diseases (EID) including COVID-19 caused by the SARS-CoV-2 virus, and the technological advancements pivotal to the improved responses—including the use of the advanced delivery technology, DNA-encoded cytokine/mucosal adjuvants, and innovative concepts in immunogen design. With continuous advancement over the past three decades, the DNA approach is now poised to develop vaccines against COVID-19, as well as other EIDs.

## Introduction

Vaccination is an extremely important approach that has impacted global health for the past centuries (1). DNA vaccines are considered as a third-generation vaccine approach that was first brought to the attention of scientific community in the early 1990s (2). As compared to vaccination approaches (recombinant proteins and viral vector), DNA plasmids encoding antigen transgenes can be rapidly and cost-efficiently manufactured (3, 4). Simple DNA plasmids do not provoke antigen specific immunity against the DNA backbone, enabling vaccine boosting in the same individuals with the same plasmid vector, and focusing the host immunity on the transgene (5). Synthetic DNA is highly stable, thereby obviating the need for cold chain transport or storage and facilitating global deployment of the vaccines during outbreaks (3, 6).

First-generation DNA vaccines had limited immunogenicity in larger mammals (NHPs) and in humans, impeding early enthusiasm for this approach (7, 8). Since these early studies, several strategies have been attempted to improve the overall immunogenicity of DNA vaccines. One such strategy is the co-delivery of DNA-vaccine with DNA-encoded cytokine adjuvant (such as IL-12) to enhance co-stimulation of the antigen-presenting cells (APCs) (9). Another important approach was the delivery of DNA vaccines alongside with adaptive electroporation (10, 11). Membrane electrochemical permeabilization and electric field created by applied voltages can significantly improve uptake of DNA plasmids into the transfected cells, improving transfection efficiency by up to 1,000-fold (12, 13). DNA vaccines, in conjunction with the newer adaptive electroporation technologies, have now been observed to induce more potent and consistent responses in a series of clinical studies against Zika, Ebola, MERS and HIV-1 [(14–17); Table 1].

**Table 1:** Review of some recent clinical trials on DNA vaccines against viral infectious diseases.

**Disease area**	**Product name**	**Vaccine antigen**	**Clinical status**	**Clinical trial number**	**Delivery method**	**Sponsor**	**Results reported**	**Humoral responses**	**Cellular responses**	**References**
HIV	PENNVAX^®^-GP	HIV Gag, Pol, Env^+/−^ pIL-12	Phase I	NCT00991354	IM-EP	Inovio Pharmaceuticals	Yes	11.1% subjects developed neutralizing antibody responses against MW965.26 by the third vaccination	88.9% subjects with either CD4 or CD8 T-cell responses by the third vaccination	(17)
	PENNVAX^®^-GP	HIV Gag, Pol, Env + pIL-12	Phase I	NCT02431767	IM-EP or ID-EP	Inovio Pharmaceuticals	Yes	Greater than 90% subjects developed binding antibody responses across groups; 75 and 50% subjects in the ID+IL12 and IM+IL12 developed neutralizing responses against MW965.25, respectively	96% subjects developed CD4 T-cell responses in ID+IL12 and IM+IL-12 groups; CD8+ T-cell responses were 64 and 44% in those groups, respectively	(18)
ZIKV	GLS5700	Consensus ZIKV prME	Phase I	NCT02809443	ID-EP	GeneOne Life Science; Inovio Pharmaceuticals	Yes	100 and 62% subjects developed binding and neutralizing antibody responses by the third vaccination, respectively; Passive transfer of Week 14 post-immune sera from 92% subjects conferred protection to mice in viral challenge	Increase in median peptide-specific IFNg responses in PBMCs following the second and third vaccinations	(14)
	VRC5283	Wildtype ZIKV prME	Phase I	NCT02996461	Needle and syringe or needle free injection	NIAID	Yes	Neutralizing antibody responses induced in 100% subjects by needle free injection system with GMT of 304	0.08 and 0.09% increase in CD4+ and CD8+ T-cell responses against E peptides in needle free injection group	(19)
Avian influenza	VRC-AVIDNA036-00-VP	H5N1 Hemagglutinin	Phase I	NCT00408109 NCT00489931	IM or ID	NIAID	Yes	At 1 mg dose, 20 and 80% subjects developed HA-binding antibody responses in the IM and ID groups, respectively	At 1 mg dose, 50 and 10% subjects developed T-cell responses by ELIspot in the IM and ID groups, respectively	(20)
EBOV	INO-4201 and INO-4202	EBOV Glycoprotein^+/−^ pIL-12	Phase I	NCT02464670	IM-EP or ID-EP	Inovio Pharmaceuticals	Yes	100 and 53% sero-conversion observed in ID-EP and IM-EP groups, respectively by the second vaccination; 38% subjects in ID-EP group developed neutralizing antibody responses by the third vaccination	An average T-cell responder rate of 74.6% observed across all cohorts	(15)
MERS	GLS-5300	MERS S protein	Phase I	NCT02670187	IM-EP	GeneOne Life Science; Inovio Pharmaceuticals	Yes	94 and 50% subjects developed binding and neutralizing antibody responses by the third vaccination	76% Subjects developed T-cell responses by the third vaccination	(16)
HTNV/PUUV	pWRG/HTN-M(x) and pWRG/PUUV-M(s2)	HTNV/PUUV Gn and Gc	Phase I	NCT01502345	IM-EP	U.S. Army Medical Research and Development Command	Yes	64 and 75% subjects receiving at least two vaccinations against HTNV and PUUV developed neutralizing antibody responses to respective viruses	Not reported	(21)
CMV	VCL-CB01 (TransVax)	CMV pp65 and gB	Phase I	N.A.	DNA vaccines formulated with poloxamer CRL1005 and benzalkonium chloride	Astellas Pharma Inc.	Yes	22.7% CMV sero-negative patients and 0% sero-positive patients have gB-specific antibody responses	36.4% CMV sero-negative patients and 22.7% sero-positive patients have pp65-specific T-cell responses	(22)
			Phase II	NCT00285259			Yes	CMV-seropositive patients undergoing hematopoietic stem cell transplants were enrolled; higher geometric mean binding titers to CMV gB detected in vaccinated groups (though not statistically significant)	Higher CMV pp65 specific IFNg T-cell responses in vaccinated patients	(23)

As with other vaccine platforms, routes of immunization can significantly impact the immunogenicity and tolerability profiles of vaccines (24). Additional advances have been reported in the latest intradermal electroporation (ID-EP) DNA delivery technology (25). As compared to conventional DNA delivery by intramuscular electroporation (IM-EP), ID-EP was observed to be well-tolerated and dose-sparing, especially in the induction of humoral immune responses (15, 26). Additionally, there has also been a recent demonstration that DNA/EP can enable direct *in vivo* production of more complex and potent nanoparticle vaccines to elicit rapid sero-conversion and more potent immune responses in animal models (27). With 30 years of development, significant technological advancements have accrued in the field of DNA vaccinology, making it an attractive approach in our efforts to develop vaccines against COVID-19 and other EIDs at the pandemic speed.

## DNA Vaccines Against EID

Between 1940 and 2004, over 300 new pathogens have been discovered, the majority of which originated from animals and were transmitted to humans by disease vectors (insects, birds and rodents) (28). Several of these, including Zika and SARS, caused regional epidemics or global pandemics, highlighting how the spread of EIDs can be compounded by global travel (29). Nucleic acid-based vaccinations (DNA and mRNA vaccines) represent an elegant approach for rapid development of vaccines against EID (30, 31). Unlike other vaccine platforms (such as protein or viral-vectored vaccines), nucleic acid-based vaccines do not require prior knowledge on production and purification of the vaccine antigens (3). Production of DNA plasmids, in particular, is relatively cost effective and straightforward, and does not require specialized pipelines (32). In theory, a vaccine can be rapidly designed so long as the protein sequences of the vaccine targets are known. Additionally, owning to the relative lack of plasmid size restriction, several vaccine antigens might be simultaneously encoded in a single DNA plasmid for explorative studies, even if the disease target is not clearly identified (33). DNA vaccines, being capable of inducing both antibody and CD8+ T-cell responses (4), may represent an attractive strategy to prevent disease transmission or improve patient clinical outcomes depending on the goals (34–36). This section will briefly review the clinical data for DNA vaccines against Ebola virus (EBOV), Zika virus (ZIKV), and Venezuelan equine encephalitis virus (VEEV), and also preclinical data for a DNA vaccine against Lassa Virus (LASV).

EBOV is the causative agent for Ebola Virus Disease (EVD), in which subjects first develop mild symptoms including fever, dysphagia, myalgia, nausea, and emesis, followed by more severe symptoms including bleeding from orifices and fulminant hepatic and renal failure, with a median mortality rate of around 50% (37). Several vaccine candidates have been developed to target EVD, including the adenovirus- and protein-based vaccine candidates, as well as FDA-approved ERVEBO-vaccine from Merck & Co, which consists of Vesicular Stomatitis Virus (VSV) live-attenuated vector modified to express EBOV glycoproteins (GP) (38). However, adverse effects such as arthralgia, lymphopenia, neutropenia related to the vector backbones have been reported in some vaccinees in early trials (39). In the DNA space, Patel et al. reported that a DNA-encoded synthetic consensus (SynCon) vaccine against EBOV GP could confer rapid protection following a single vaccination in mice from a heterologous mouse-adapted challenge strain (40). In NHPs, a two-dose dose-sparing ID SynCon DNA vaccine regimen conferred 100% protection from challenge, inducing durable responses in the animals a year after the final vaccination (40). In a first-in-human (FIH) DNA vaccine study, the aforementioned SynCon EBOLA GP vaccine (INO-4201) as well as another DNA vaccine encoding EBOV-GP from a 2014 outbreak Zaire Makona strain (INO-4202) were given individually, or in combination along with a DNA-encoded human IL-12 adjuvant in a three-dose regimen delivered by IM- or ID-EP (15). Robust antibody responses were induced in every arm. In particular, the ID-EP delivery of INO-4201 induced extremely rapid sero-conversion, with 100% sero-activity observed in all participants by the second vaccination. T-cell responses were observed in 70% of participants overall. Cytokine responses were detected in peripheral blood CD8+ and CD4+ T-cells, especially in subjects receiving ID DNA vaccination. Durable responses were observed in most participants, with a Geometric Mean Titer (GMT) of 42 in the ID INO-4201 group 48 weeks after the final vaccination (15).

ZIKV is a mosquito-borne illness for which patients can present with fever, malaise, rash and conjunctivitis. Additionally, ZIKV infection in pregnant women can cause significant birth defects including microcephaly and in men can cause testicular atrophy (41, 42). In preclinical studies, DNA vaccination with ZIKV pre-membrane + envelope proteins (prME) induced robust humoral and cellular responses in both mice and non-human primates (NHPs) (43, 44). In a study where mice were vaccinated intramuscularly with a ZIKV prME DNA vaccine without EP, both antibody and T-cell responses were induced, though the magnitudes were lower than those induced by a viral vectored ChAdOX1 vaccine (45). IM-EP-mediated vaccination of a different ZIKV DNA vaccine (GLS-5700) in IFNAR-/- mice decreased brain viral load and protected them from weight loss following lethal ZIKV challenges. Passive transfer of sera from GLS-5700 vaccinated NHPs similarly protected the IFNAR-/- mice in challenges (44). In a separate NHP study, two-dose intramuscular DNA vaccination of wildtype ZIKV prME (VRC5283) induced potent binding and neutralizing antibodies at both 1 and 4 mg doses and conferred complete protection to macaques from subsequent ZIKV challenges (43). Promising results have also been observed in several clinical ZIKV DNA vaccine studies. Tebas et al. reported that the three-dose ID DNA vaccination of GLS-5700 was safe and effectively elicited binding antibody responses in 100% of participants and 63% neutralizing antibody responses by the third vaccination (14). Passive transfer of post-immune sera from the participants to IFNAR-/- mice effectively protected them from lethal ZIKV challenges. T-cell responses were also induced in the subjects, particularly in subjects receiving the higher dose (2.0 mg), with a median IFNγ ELIspot counts of 58 per million PBMCs at 2 weeks post the second vaccination (as compared to 0 spots per million PBMCs observed at baseline) (14). In a separate Phase I DNA vaccine trial, participants received a three-dose DNA vaccine regimen at 4 mg dose against wildtype ZIKV prME (VRC5283) via a needle-free injection system. Sero-conversion was observed in 100% participants by the third vaccination with a neutralization GMT of 304. Relative to baseline, there was an increase in cytokine responses by 0.08 and 0.09% in peripheral blood CD4+ and CD8+ T cell, respectively (19).

VEEV is a mosquito-borne alphavirus that can cause febrile illness and progressive encephalitis in both equines and humans. Currently, there is no FDA-approved vaccine or immunotherapy against VEEV (46). Hannaman et al. reported a Phase I DNA vaccine encoding the VEEV E3-E2-6K-E1 genes (47). Subjects received a three-dose DNA vaccine regimen delivered by either IM- or ID-EP with the Ichor Delivery System. The vaccine was safe and well-tolerated. Neutralizing antibody responses were observed in all volunteers receiving the IM DNA vaccine, and 87.5 and 62.5% subjects receiving the high dose or low dose ID DNA vaccine respectively, even though the dosage used in the ID vaccinations were lower than those in the IM vaccinations (DNA doses of 0.08 or 0.3 mg for ID vs. 0.5 or 2.0 mg for IM). In this small study, robust and durable neutralizing antibody responses were observed in the high dose IM group a year after the initial vaccination (47).

There are additional preclinical studies describing the design and evaluation of DNA vaccines against other EIDs. Lassa fever, caused by LASV, is endemic in West Africa and patients can present with hemorrhages from orifices, respiratory distress and shock, and is associated with a significant mortality rate of 80% (48). Jiang et al. reported that DNA vaccines against LASV glycoprotein precursor gene, generated robust humoral and cellular immunity in both guinea pigs and NHPs and completely protected them from viremia, clinical disease, and death following lethal LASV challenges (26).

## DNA Vaccines Against SARS-CoV and MERS-CoV

Coronaviruses are a group of enveloped positive-sense RNA viruses that can cause mild to severe respiratory infections in mammals and birds (49). While they are used to be associated with milder infections such as the common cold, three variants- Severe Acute Respiratory Syndrome Coronavirus (SARS-CoV), Middle East Respiratory Syndrome Coronavirus (MERS-CoV) and SARS-CoV-2- are associated significant morbidity and mortality in the infected individuals, causing major regional epidemics or global pandemics in the twenty-first century (50). While several vaccine candidates appear promising in preclinical animal studies and are currently being evaluated in the early phase clinical trials, there is to date no approved vaccine against these coronaviruses, highlighting the urgent need for rapid and successful development of effective vaccines to mitigate the global outbreaks (51).

SARS-CoV is the virus responsible for the SARS outbreak which originated in Guangdong, China, and subsequently spread globally to affect countries in Southeast Asia, North and South America, and Europe, with a cumulative case counts of at least 8,000 and a global death toll of at least 774 (52, 53). The virus was transmitted zoonotically from a civet cat to a human (54). Following the outbreak in 2002-2003, a preclinical SARS-CoV DNA vaccine was reported in 2004. DNA vaccine against SARS-CoV S protein administered intramuscularly by needle and syringe induced both neutralizing antibody and also T-cell responses in mice (34). Vaccinated mice had six orders of magnitudes lower lung viral load following intranasal challenge. T-cell depletion and serum passive transfer studies demonstrated that protection against SARS-CoV in vaccinated mice depended only on serum antibodies but not on T cells (34). This vaccine candidate was evaluated in a Phase I trial between 2004 and 2005 (55). Subjects received a three-dose IM DNA vaccine regimen at the 4 mg dose. Binding and neutralizing antibody responses were detected in 80 and 50% of vaccinated individuals, respectively. CD4+ T-cell responses were detected in all vaccinees, whereas CD8+ T-cell responses were detected in 20% of subjects. This study showed 30% vaccinees remained sero-positive 24 weeks following the final vaccination.

MERS-CoV was responsible for the local outbreak of MERS in the Arabian Peninsula in 2012, and the local epidemic in Seoul, South Korea, in 2015 (56). MERS-CoV is considered as a zoonotic virus with a mortality rate of 35%, transmitted originally from an infected camel to a human (57). Muthumani et al. described the development of DNA vaccine encoding MERS spike protein, which induced potent humoral and cellular responses in mice, camels and NHPs. NHPs receiving the IM DNA vaccines were significantly protected in the subsequent MERS challenge, having lower viral load, fewer clinical symptoms and lung pathology as compared to the control macaques. Importantly upon challenge, histological examinations did not reveal any signs of disease enhancement in macaques receiving either high or low dose DNA vaccines (58). This DNA vaccine (GLS-5300) was subsequently evaluated in a Phase I dose-escalation study. Subjects received three IM vaccinations at the low (0.67 mg), medium (2.0 mg), and high (6.0 mg) doses (16). The vaccines were observed to be safe and well-tolerated, and induced sero-conversion in 94%, neutralizing antibody responses in 50%, and T-cell responses in 50% individuals by the third vaccination. Importantly, 48 weeks post the final vaccination, humoral, and cellular responses were still observed in 77 and 64% of subjects, respectively (16).

## Developing DNA Vaccines Against SARS-CoV-2

### Overview of SARS-CoV-2

SARS-CoV-2 is the virus responsible for causing COVID-19, which swept across the globe to affect over 200 countries between 2019 and 2020, and is associated with more than 13 million cases globally and a death toll over 550,000 as of July 13th, 2020 (59, 60). Similar to SARS-CoV, SARS-CoV-2 gains entry into the target cells through viral S protein and host ACE-2 interaction. Priming of the viral S protein through proteolytic cleavage with host serine protease TMPRSS2 is also known to be important for viral entry (61, 62). ACE-2 is known to be abundantly expressed in many tissues, including small intestines, kidneys, and cardiac muscles (63). Clinically, COVID-19 patients can present with cardiac failure, hepatic and renal failure, as well as acute respiratory distress syndrome, and have an estimated disease mortality rate of approximately 1.3% (64). Other than significant morbidity and mortality associated with the global pandemic, efforts to mitigate the virus, including social distancing and lockdown, have significantly disturbed the global economic activity (65). There is an urgent need to develop effective and sustainable approaches to contain the global spread of the virus.

### Vaccine Strategies Against SARS-CoV-2

Development of effective vaccines against SARS-CoV-2 is considered to be one of the most important mitigation measures (51). In the NHP model, prior infection has been observed to protect animals from subsequent SARS-CoV-2 viral exposure (66, 67). Several vaccine strategies against SARS-CoV-2, including RNA vaccines (68–71), DNA vaccines (72, 73), inactivated virus vaccines (74), viral vectored vaccines (75), and recombinant protein vaccines (76), are concurrently being explored and are at different stages of clinical development (77). Most vaccine candidates target the SARS-CoV-2 spike (S) protein (78). The mRNA vaccine candidate mRNA-1273, in particular, has demonstrated protective efficacy in mice challenged with mouse-adapted SARS-CoV-2 virus (68) and has also been shown to induce consistent binding and neutralizing antibody responses in vaccinated individuals (70); whereas the inactivated vaccine PiCoVacc and viral-vectored vaccine ChAdOx1 nCoV-19 demonstrated complete and partial protection in rhesus macaques challenged with SARS-CoV-2 (74, 76). Importantly, while antibody-dependent enhancement (ADE), a process in which non-neutralizing antibodies induced in a host from vaccination or prior infection subsequently exacerbate viral infection in the host from a second exposure, had been observed in macaques vaccinated with a MVA-vectored SARS-CoV vaccine and challenged with SARS-CoV (79), it had not been observed in PiCoVacc or ChAdOx1 nCoV-19 vaccinated macaques that were subsequently challenged with SARS-CoV-2 (74, 76). At present, there are no known clinical findings that differentiate severe viral infections from immune-enhanced diseases (80, 81).

In terms of immune correlates of protection, neutralizing antibodies (NAbs) are considered important in preventing transmission of SARS-CoV-2. For example, passive transfer of a human SARS-CoV-2 neutralizing antibody from a convalescent patient into Syrian hamsters was observed to confer protection from SARS-CoV-2 exposure in a dose-dependent manner (82). Increasingly, however, there is an appreciation of the importance of cellular immunity in the resolution of SARS-CoV-2 infection. Two patients with agammaglobulinemia, for example, successfully recovered from COVID-19 (83). Additionally, other serological surveys of convalescent COVID-19 patients demonstrate that there is a surprisingly large proportion of recovered patients without robust neutralizing antibody titers during the early convalescent phase (84). Additionally, unexpectedly, higher titers of neutralizing antibodies were observed in patients with more severe disease (85). In critically ill patients, it was observed that CD4+ T-cell responses were relatively impaired, while IgG antibody responses were surprisingly robust (86). These studies highlight the importance of developing vaccine approaches that can induce both humoral and cellular immunity, and the need for continuous ongoing efforts to monitor the vaccine safety profiles during the current pandemic.

### DNA Vaccines Against SARS-CoV-2 Currently Under Development

Recently, Smith et al. reported rapid development of a DNA vaccine candidate (INO-4800) against the SARS-CoV-2 S protein. The design of this vaccine candidate leveraged previous understanding of SARS-CoV and MERS-CoV S protein folding. INO-4800 induced robust binding and neutralizing antibody responses as well as antigen-specific T-cell responses in both mice and guinea pigs (72). The vaccine candidate also induced strong binding and neutralizing antibody responses as well as T-cell responses in an NHP study at both 1 and 2 mg doses (87). When the macaques were challenged 3 months post-vaccination, they developed significantly lower lung viral load and faster viral clearance in the nose as compared to control macaques. Additionally, vaccinated macaques were observed to have fast recall responses, in which binding and neutralizing antibody titers rise rapidly 7 days post-viral inoculation, as compared to control macaques (87). In a parallel study, Yu et al. demonstrated in an NHP challenge model that two intramuscular DNA vaccinations at 5 mg dose of variants of SARS-CoV-2 S protein induced binding and neutralizing antibody responses, CD4+ and CD8+ T-cell responses, and decreased viral shedding when macaques were challenged (73). Vaccine induced neutralizing antibodies at the time of challenge was observed to be strongly correlated with challenge protection. Of note, INO-4800 is currently being evaluated in a Phase I clinical study (NCT04336410) in which subjects received two EP-mediated ID DNA vaccinations at the low (1.0 mg) or regular (2.0 mg) doses in the US. Concurrently, the vaccine candidate will be evaluated in a Phase I/IIa study in the Republic of Korea (NCT04447781). Safety and immunogenicity data from this trial will provide valuable insights to understand and evaluate the DNA vaccine approach against SARS-CoV-2 during the current pandemic.

## Latest Advancement in the DNA Vaccine Technology

The observation that DNA vaccines induce more potent and consistent responses in clinical trials can be attributed to the recent advances in synthetic DNA and electroporation technology. In fact, enhanced DNA/EP parameters have also allowed biologics to be delivered systematically to achieve potent and durable *in vivo* expression in animals (88–91). An DNA-encoded Monoclonal Antibody (DMAb) against Zika virus, in particular, is currently under clinical evaluation in a Phase I study (NCT03831503). The next section will highlight some additional recent developments in DNA vaccine technology.

### DNA-Encoded Cytokine Adjuvants

Several studies have reported that incorporating DNA-encoded cytokines with DNA vaccines could adjuvant vaccine induced responses (6). Co-delivery of IL-2, for example, was observed to improve the immunogenicity of DNA vaccines against SARS-CoV S and N proteins, influenza H1N1 hemagglutinin and neuraminidase, and HIV gp120 and Nef (92–94). Co-delivery of DNA vaccine against liver-stage malaria antigens with IL-33 was found to improve liver-localized CD8+ T-cell responses and confer improved protection to mice from Plasmodium challenge (95). Co-administration of IL-36γ, on the other hand, was found to improve immune responses induced by DNA-encoded Zika, HIV and influenza vaccines, and reduced dose requirement of DNA vaccines in mice to protect against ZIKV challenge (96). Co-delivery of DNA vaccines with plasmid-encoded IL-12 (pIL-12) in particular has drawn significant interests recently. The importance of IL-12 in inducing Th1-biased immunity and augmenting CD8+ cytotoxic T-cell activity is well-established (97, 98). In preclinical studies, plasmid-encoded IL-12 was found to increase IFNγ+ T-cell responses induced by DNA vaccines against HIV, HCV, HSV-2, and *Toxoplasma gondii* (98–101). In a Phase I study (HVTN098), coformulation of PENNVAX^®^-GP with pIL-12 significantly increased the proportion CD4+ T-cell responders from 56 to 96% (18).

### Targeted DNA Vaccine Delivery

Intracellular barriers can negatively impact expression and presentation of a DNA-encoded antigen (102). Approaches to enhance targeting of DNA-encoded transgene to the desired cellular compartments have been quite successfully explored. Given the observation that secreted antigens elicit enhanced antigen-specific antibody responses than cytosolic antigens in some studies (102), leader sequences have been designed and used to facilitate secretion of DNA-encoded antigens. The incorporation of a tissue plasminogen activator (TPA) leader sequence, for example, has been shown to enhance antibody responses, cellular responses, and protection against mycobacterial antigens (103), and also increase antibody responses to a major birch pollen antigen (104). The use of human IgE leader sequence has similarly been demonstrated to improve trafficking of a DNA-encoded transgene to the secretory network (88). In the scenarios where the induction of CD8+ T-cell responses are preferred, DNA vaccines are designed to encode antigens fused to ubiquitin (102). Ubiquitin fusion facilitates proteasomal turnover of the tagged antigens into epitope peptides and enhances targeting of the antigens to the MHC Class I pathway. This strategy has been explored for several disease targets, including LCMV, influenza and melanoma (105–107). All studies consistently demonstrated that ubiquitin-conjugation enhanced CTL responses. Interestingly, antibody responses were observed to simultaneously decrease (102, 106). In other scenarios where the induction of CD4+ T-cell responses and humoral responses are desired, DNA-encoded antigens are designed to incorporate a lysosomal targeting moiety, given the abundance of MHC Class II complexes in the cellular endosomal/lysosomal compartment (108). Linkage of antigen to Lysosomal Associated Membrane Protein type 1 (LAMP-1), for example, has been found to increase the DNA-vaccine induced neutralizing antibody responses against Dengue virus (109) and the binding antibody responses against West Nile virus (110). It can be envisioned that the immune responses induced by a SARS-CoV-2 DNA vaccine may be further tailored with the aforementioned approaches.

### Intradermal vs. Intramuscular DNA Vaccination

In terms of DNA vaccine delivery, the ID-EP technology has recently drawn significant interests. This approach harnessed the biology of antigen presenting cells, enriched in human skin tissues, for the induction of immune responses (111). Delivery of DNA vaccines by ID-EP was observed to result in direct transfection of dermal dendritic cells (DCs), which subsequently migrated to the draining lymph nodes (112). In rats, single delivery of low-dose DNA vaccine against RSV F protein by ID-EP conferred complete protection from RSV/A challenge (113). In comparison, intramuscular DNA vaccination can result in efficient transfection of myocytes, which may in turn secrete soluble antigen through the lymphatic drainage systems (6). Additionally, especially in the context of electroporation, DNA vaccination can result in formation of apoptotic muscles cells harboring plasmid-encoded antigens, which can then be taken up by muscle-infiltrating APCs for cross presentation (6). ID DNA vaccines were found to be more well-tolerated than IM DNA vaccines in humans. In terms of induction of humoral responses, some preliminary studies suggest that ID vaccination enhanced humoral responses and was dose sparing, with or without EP (20, 47, 114). In terms of cellular responses, IM DNA vaccination was observed to induce stronger T-cell responses than ID DNA vaccination in some studies (20), and similar responses in other studies (18). In the Phase I EBOV DNA vaccine study, ID-EP vaccination of INO-4201 induced 100% sero-conversion post-dose two, whereas 53% sero-conversion was induced by the IM delivery of INO-4201. Upon the completion of the 3-dose regimen, overall T-cell responses were induced in 53.3% subjects receiving IM INO-4201 and 73.3% subjects receiving ID INO-4201 (15). In the Phase I HIV DNA vaccine study (HVTN098), ID delivery of PENNVAX^®^-GP at a lower dose of 1.6 mg elicited higher magnitude of gp140-specific binding antibody responses in subjects than IM delivery of the vaccine at 8.0 mg dose. In terms of cellular responses, 96% CD4+ T-cell response rates were observed in both ID+IL12 and IM+IL12 groups, whereas 64 and 44% CD8+ T-cell response rates were observed in the ID+IL12 and IM+IL12 groups, respectively (18).

### Intranasal DNA Vaccination and Other Strategies to Enhance Mucosal Immunity

Transmission of several viral pathogens, such as HIV-1, influenza and SARS-CoV-2, can occur through mucosal sites (115–117). Strategies to enhance mucosal immunity for DNA vaccines, therefore, can be important. Secretory IgAs play a special role in host mucosal defense. As compared to IgG, IgA is typically 30–100 times more concentrated at the mucosal site, due to its ability to resist protease degradation (118). Intranasal DNA vaccination is one such strategy to promote trafficking of antigens to mucosal associated lymphoid tissue to prime mucosal immunity. In one study, it was observed that an intranasal (IN) H5N1 HA DNA vaccine co-administered with PEI induced comparable serum HAI titers as compared to an ID H5N1 DNA vaccine, but the IN vaccination elicited significantly higher HAI titers in Bronchoalveolar lavage (BAL) fluid as well as higher serum and BAL IgA levels (119). In a separate study, Kumar et al. reported the use of chitosan nanoparticles to intranasally deliver DNA vaccine against acute respiratory syncytial virus (RSV) infection. High levels of serum IgG and mucosal IgA antibodies were induced following mucosal vaccination. Strong CD8+ T-cell responses were also induced systemically and in the lung following viral challenge (120). While promising, this approach may be associated with certain pitfalls. Certain agents used to facilitate mucosal DNA delivery, such as PEI, are non-biodegradable and can be associated with significant toxicity (121). Additionally, there may be concerns and required additional safety studies to investigate the effects of targeting the nasal mucosa, considering the olfactory epithelium is the only part of the central nervous system (CNS) exposed to the external environment. Components of intranasal vaccines may therefore gain easy access to the CNS, bypassing blood brain barrier (121). In light of these challenges, alternative approaches can be considered to increase mucosal responses for DNA vaccines, such as through the use of mucosal adjuvants. For example, in the HIV and influenza model, combined delivery of DNA vaccines with plasmids encoding a mucosal chemokine, CCL25, was found to increase antigen-specific responses in the lung and mesenteric lymph nodes, and also increase antigen-specific CD4+, CD8+ T-cell responses as well as IgA responses at the mucosal sites (122). Additionally, in the NHP model, co-administration of an SIV DNA vaccine with plasmid-encoded CCR10L increased serum and vaginal IgA levels and conferred improved protection to macaques from SIVsmE660 challenge (123). Further exploration of approaches that may adjuvant mucosal responses of a SARS-CoV-2 DNA vaccine is likely of relevance.

### DNA Launched Nano-Vaccines

While genetic adjuvants have been demonstrated to enhance immunogenicity of DNA vaccines in several cases, their use is highly contingent upon correct dose titration for adjuvants vs. vaccines in both animals and humans (124). Additionally, each plasmid-encoded adjuvant will likely need to undergo additional preclinical toxicology evaluation (125). The use of DNA-launched nano-vaccine technology to enhance DNA vaccine responses is potentially a viable and convenient alternative to these challenges. As compared to monomeric antigens, nanoparticle vaccines have been demonstrated to induce significantly stronger humoral responses to various targets including HIV, influenza, RSV, and malaria (126–130). However, assembly of these nano-vaccines is technologically cumbersome and costly with the conventional techniques (131). Xu et al. recently demonstrated that DNA/EP can enable direct *de novo* assembly of nanoparticle vaccines in the hosts to bypass such complex production processes (27, 132). As compared to DNA-encoded monomeric vaccines, DNA-launched nano-vaccines induced more rapid sero-conversion, higher binding, and neutralizing antibody titers, stronger CD8+ T-cell responses and conferred improved protection in a mouse influenza challenge model in a dose-sparing fashion and additional study is warranted (27, 133).

## Concluding Remarks

This paper reviewed some major advancements in the DNA vaccine field, which support the findings that the newer DNA vaccines are now inducing more potent, consistent, and durable immune responses in several recent clinical studies. Advances in synthetic DNA and EP technology were harnessed for rapid design and evaluation of a novel DNA vaccine against SARS-CoV-2. Within 10 weeks from publication of the viral sequences, the vaccine is now being evaluated in a Phase I clinical study. The Phase I study was expanded to included older participants (134). It remains to be explored how the immune outcomes induced in the older participants compared to younger participants, as it has previously been observed that less potent humoral responses were observed in older volunteers by a mRNA vaccine candidate, and for protein based approaches higher antigen doses can improve immune responses in this population (135).

Another foreseeable challenge is the durability of vaccine responses. Immune responses against SARS-CoV-2 in natural infection were reported to taper relatively quickly. In one study, 40% of asymptomatic patients become seronegative for N protein and an S2 peptide during early convalescence (136). In a separate study, antibody titers in exposed healthcare workers were observed to quickly decline over two-month period (81). For DNA vaccines, some early clinical studies found that the vaccine responses were more limited in durability. For a SARS DNA vaccine candidate, seropositivity rate declined from 80% at the peak to 30% 24 weeks following the final vaccination (34). In some more recent studies, durability was found to be improved. For a MERS DNA vaccine candidate, seropositivity rate declined from 94% at the peak to 77% 48 weeks post the final vaccination, while the cellular responses increased from 50% during the final vaccination to 64% 48 weeks post the final vaccination (16). It will therefore be pertinent to evaluate the durability of vaccine responses of INO-4800 and other SARS-CoV-2 vaccine candidates in comparison with the kinetics of immune responses induced following a natural SARS-CoV-2 infection. Furthermore, milligram levels of DNA are currently required in DNA vaccine regimen. While DNA can be quickly and inexpensively manufactured, studies to further reduce the dose are of continued importance.

Finally, another important consideration for DNA vaccination is the heterologous DNA prime protein/viral vector boost strategy, which has been extensively explored for many disease targets, including HIV (137, 138), influenza (139, 140), malaria (141), and tuberculosis (142), and has been demonstrated to improve vaccine immunogenicity and protection in many cases (143). The utility of the DNA platform to boost many other platforms in a highly tolerable approach could be advantageous for expanding immunity and memory responses from other vaccine platforms.

In summary, owning to the strong clinical safety profile, low costs of production and transportation, and the unique ability to induce both humoral and cellular responses, newer DNA vaccine will be an extremely important tool as part of the current COVID-19 pandemic and will possibly help address other EIDs. Continuous development in this technology, especially in such areas as DNA-encoded cytokine adjuvants, DNA-launched nano-vaccines, advanced plasmid delivery technologies, and innovative prime-boost strategies will have significant impact on continually advancing this platform to impact human and animal health.

## Author Contributions

ZX and DW conceptualized the paper. ZX, AP, NT, XZ, KM, DK, and DW wrote the paper. All authors contributed to the article and approved the submitted version.

## Conflict of Interest

ZX, DW, and DK have a pending patent US.62784318. KM receives grants and consulting fees from Inovio related to DNA vaccine development. DW has received grant funding, participates in industry collaborations, has received speaking honoraria, and has received fees for consulting, including serving on scientific review committees and board series. Remuneration received by DW includes direct payments, stock or stock options, and in the interest of disclosure he notes potential conflicts associated with his work with Inovio and possible others. The remaining authors declare that the research was conducted in the absence of any commercial or financial relationships that could be construed as a potential conflict of interest.
